# Zoeppritz-based AVO inversion using an improved Markov chain Monte Carlo method

**DOI:** 10.1007/s12182-016-0131-4

**Published:** 2016-12-20

**Authors:** Xin-Peng Pan, Guang-Zhi Zhang, Jia-Jia Zhang, Xing-Yao Yin

**Affiliations:** 1 School of Geosciences, China University of Petroleum (Huadong), Qingdao, 266580 Shandong China; 2 Laboratory for Marine Mineral Resources, Qingdao National Laboratory for Marine Science and Technology, Qingdao, 266071 Shandong China

**Keywords:** Adaptive Metropolis (AM) algorithm, Delayed rejection (DR) algorithm, Bayesian AVO inversion, Exact Zoeppritz, Nonlinear inversion

## Abstract

The conventional Markov chain Monte Carlo (MCMC) method is limited to the selected shape and size of proposal distribution and is not easy to start when the initial proposal distribution is far away from the target distribution. To overcome these drawbacks of the conventional MCMC method, two useful improvements in MCMC method, adaptive Metropolis (AM) algorithm and delayed rejection (DR) algorithm, are attempted to be combined. The AM algorithm aims at adapting the proposal distribution by using the generated estimators, and the DR algorithm aims at enhancing the efficiency of the improved MCMC method. Based on the improved MCMC method, a Bayesian amplitude versus offset (AVO) inversion method on the basis of the exact Zoeppritz equation has been developed, with which the P- and S-wave velocities and the density can be obtained directly, and the uncertainty of AVO inversion results has been estimated as well. The study based on the logging data and the seismic data demonstrates the feasibility and robustness of the method and shows that all three parameters are well retrieved. So the exact Zoeppritz-based nonlinear inversion method by using the improved MCMC is not only suitable for reservoirs with strong-contrast interfaces and long-offset ranges but also it is more stable, accurate and anti-noise.

## Introduction

Inversion of reflection coefficients extracted from amplitudes of seismic waves can provide estimates of significant reservoir parameters. In many cases, the approximations of Zoeppritz equation at small and moderate incidences are sufficiently accurate to reproduce the exact reflection coefficients quantitatively (Jilek 2001; Buland and Omre 2003; Zhang et al. 2011, 2014a, b; Russell et al. 2011; Zong et al. 2012, 2013a, b; Yang et al. 2015). In terms of the reservoirs with strong-contrast interfaces (salt domes, heavy oil fields, basalts, etc.) and long-offset ranges, however, the conventional AVO inversion based on the approximations would be not appropriate or suitable (Larsen 1999; Chen et al. 2009; Liu et al. 2010, 2012a, b, 2014; Wang et al. 2011; Zhu et al. 2012; Zhang et al. 2013; Huang et al. 2013; Zhi et al. 2013, 2015; Zong et al. 2013a, b; Lu et al. 2015; Lehochi et al. 2015).

A more sophisticated and time-consuming nonlinear inversion based on the exact Zoeppritz equation provides an alternative to obtain more accurate three AVO parameters. Chen and Wei (2012) studied the joint PP and PS AVO inversion based on Zoeppritz equation in ray parameter domain. Not having used PS-wave constraint, Zhu and McMechan (2012) proposed to apply the “exact” elastic Zoeppritz equations to do the AVO inversion for reflections without critical angles. Zhi et al. (2013) made efforts to explore the joint nonlinear least-squares inversion with a trust-region reflective Newton method. Based on the generalized linear inversion theory, Wang et al. (2011), Zhang et al. (2013) and Huang et al. (2013) studied the pre-stack inversion using the Zoeppritz equation. Liu et al. (2014) explored the joint AVO inversion of PP and PS reflections based on the Zoeppritz equation using the reflectivity method. Zhi et al. (2015) proposed an iterative regularizing Levenberg–Marquardt (IRLM) scheme for Zoeppritz-based pre-stack amplitude versus angle (AVA) inversion. Combining the PP and PS information based on a least-squares approach, Lu et al. (2015) developed a method of nonlinear joint pre-stack inversion for the P- and S-wave velocities and the density. Lehocki et al. (2015) proposed a method of probabilistic estimation for density and shear information from Zoeppritz’s equation, which is used for better hydrocarbon detection in sandstone reservoirs. Obviously, inverting the exact reflection coefficients based on the exact Zoeppritz equation completely removes the bias caused by the inaccuracy of the approximate reflection coefficients of Zoeppritz equation. Also, the nonlinear inversion may produce results with higher accuracy and resolution (Jilek 2002; Rabben et al. 2008; Zong et al. 2013a, b; Fernández-Martínez et al. 2013; Zunino et al. 2015), improving the accuracy of reservoir prediction and fluid identification (Tian et al. 2013; Du and Yan 2013; Yin et al. 2013; Wang et al. 2014; Yin et al. 2015). This paper is devoted to the nonlinear inversion of the exact reflection coefficients $R_{\text{PP}}$ based on the exact Zoeppritz equation to obtain the P- and S-velocities and density directly.

The core of the MCMC algorithm is Markov chains, and the convergence properties and convergence speed of Markov chains limit to the proposal distribution. In order to improve the optimization of proposal distribution in the inverse problems, especially in the high-dimensional inverse problems, Haario et al. (2001) proposed an adaptive Metropolis algorithm based on the global adaptive strategy to adaptively update the proposal distribution. In addition, the MCMC algorithm has difficulty in launching when the proposal distribution is far away from the target distribution, and then Green and Mira (2001) proposed a delayed rejection algorithm based on the local adaptive strategy, which turns out to be an effective solution to the problem. We intend to propose an improved MCMC method, combining both advantages of AM and DR algorithm, and use Bayesian theory to introduce a priori information. Ultimately, we will realize the Zoeppritz-based AVO inversion, which can estimate P- and S-velocities and density directly, and estimate the uncertainty of AVO inversion results. We will initially introduce the improved MCMC method combining the AM and DR algorithm, and then discuss the Bayesian AVO nonlinear inversion method to obtain the P- and S-velocities and density directly by using the exact Zoeppritz equation, and estimate the uncertainty of AVO inversion results based on the logging data (Hong and Sen 2009). We end with real data case studies that illustrate the method.

## Theory

### The improved MCMC method

Based on the Bayesian framework, the MCMC method uses the existing data to constrain the solutions, which not only satisfies the statistical characteristics of the inversion parameters but also integrates the prior information to improve the inversion accuracy. Moreover, the MCMC algorithm can jump out of the local optimal solutions in the optimization process to get the global optimal solution. Sampling the Bayesian posterior probability density distribution, the MCMC method can obtain mass samples, statistically analyzed to acquire the estimators indirectly and the uncertainty information as well. The principle of the conventional MCMC method is described in Zhang et al. (2011), and there is no need for it to be reiterated here.

The core of the MCMC method is Markov chains, while the convergence properties and convergence speed is subject to the shape and size of proposal distributions (Wang and Zhang 2010). In order to improve computational efficiency, the usage of an appropriate proposal distribution is necessary, especially for high-dimensional inverse problems. Meanwhile, the MCMC method is not easy to start when the initial proposal distribution is far away from the target distribution. So, we adopt an improved MCMC method, combining the AM algorithm based on the global adaptive strategy (Haario et al. 2001) and the DR algorithm based on the local adaptive strategy (Green and Mira 2001) to speed up the convergence of Markov chains.

The core of the AM algorithm is to build a Gaussian proposal distribution, and assumed at time *t* in the program, we have already created chain *q* ^0^, *q* ^1^, …, *q* ^*t*−1^. The proposal distribution is now defined as the Gaussian distribution with mean at the current state *q* ^*t*−1^ and covariance Cov(*q* ^0^, *q* ^1^, …, *q* ^*t*−1^), and the covariance is set to be:

1 $$C_{i} = \left\{ {\begin{array}{*{20}l} {C_{0} } \hfill & \quad{i \le t_{0} } \hfill \\ {s_{d} {\text{Cov}}(q^{0} ,q^{1} , \ldots ,q^{t - 1} ) + s_{d} \varepsilon I_{d} } \hfill & \quad{i > t_{0} } \hfill \\ \end{array} } \right.$$

where, *t*_0_ is the initial period after which the adaptation began; *C*_0_ is the initial covariance, which is chosen according to a priori information when *t* < *t*_0_; *s*_*d*_ is a parameter that depends only on the dimension *d*, which is often chosen to be 2.4^2^/*d* according to Gelman et al. (1996); *ɛ* > 0 is a very small constant to ensure that the covariance matrix *C*_*i*_ is not a singular matrix; *I*_*d*_ denotes the *d*-dimensional identity matrix. And after some formula manipulation, the covariance *C*_*i*+1_ satisfies the recursive formula:

2 $$C_{i + 1} = \frac{i - 1}{i}C_{i} + \frac{{s_{d} }}{i}\left( {i\bar{q}_{i - 1} \bar{q}_{i - 1}^{T} - (i + 1)\bar{q}_{i} \bar{q}_{i}^{T} + q_{i} q_{i}^{T} + \varepsilon I_{d} } \right),$$

where $\overline{q}_{i}$ denotes the mean value of the previous sampled Markov chain. So we can estimate the covariance with less computational cost by using the recursive formula (Haario et al. 2001).

The DR algorithm is an improved MCMC method, and its basic idea is allowing partial local adaptation of the rejected candidates, where the Markov chains still retain the Markovian property and converge to the second or higher stages. The ultimate targets of the DR algorithm are to improve the accuracy and efficiency of the estimators. The creation of proposal distributions in higher stages is allowed to depend not only on the current position of the chain but also on the proposal distribution created previously and the rejected candidates in higher stages (Green and Mira 2001; Haario et al. 2006). Suppose a Markov chain that has $\pi$ as its unique stationary distribution is created, and the current position of the Markov chain is *x*_*t*_, then a candidate move, x*, is generated from a proposal *q*_1_(*x*, x*) and accepted with the probability.

3 $$\alpha_{1} (x_{t} ,x^{*} ) = \hbox{min} \left\{ {1,\frac{{\pi (x^{*} )q_{1} (x^{*} ,x_{t} )}}{{\pi (x_{t} )q_{1} (x_{t} ,x^{*} )}}} \right\}$$

When rejected in first stage, a second stage move, *y*, is generated from a proposal *q*_2_(*x*_*t*_, x*, *y*). The second stage proposal is accepted with probability.

4 $$\alpha_{2} (x_{t} ,x^{*} ,y) = \hbox{min} \left\{ {1,\frac{{\pi (y)q_{1} (y,x^{*} )q_{2} (y,x^{*} ,x_{t} )\left[ {1 - \alpha_{1} (x^{*} ,x_{t} )} \right]}}{{\pi (x_{t} )q_{1} (x_{t} ,x^{*} )q_{2} (x_{t} ,x^{*} ,y)\left[ {1 - \alpha_{1} (x_{t} ,x^{*} )} \right]}}} \right\}$$

Of course, this process of delaying rejection can be iterated, and the expression in higher stages can be seen in Green and Mira (2001) and Haario et al. (2006).

It may be difficult to get the AM adaptation started when the initial proposal distribution is far from the correct one and the DR framework provides a natural remedy for these situations. The covariance at the DR stage *j* can be computed simply by scaling the covariance produced by the AM step: *C*_*i*_^*j*^ = *γ*_*i*_ *C*_*i*_, where *j* = 1… *m*. Here *m* is the number of DR stages applied for every rejected point, and *m* is often chosen to be 2 in practice (Haario et al. 2006). So the improved MCMC method enhances the efficiency compared to the conventional MCMC and the AM algorithm especially when the initial proposal distribution is badly chosen. In addition, if the algorithms have difficulties in getting themselves moving especially when the acceptance ratio resulted in MCMC method is very low, the improved MCMC method, with second stage moves scaled down, can provide help.

**Fig. 1:**
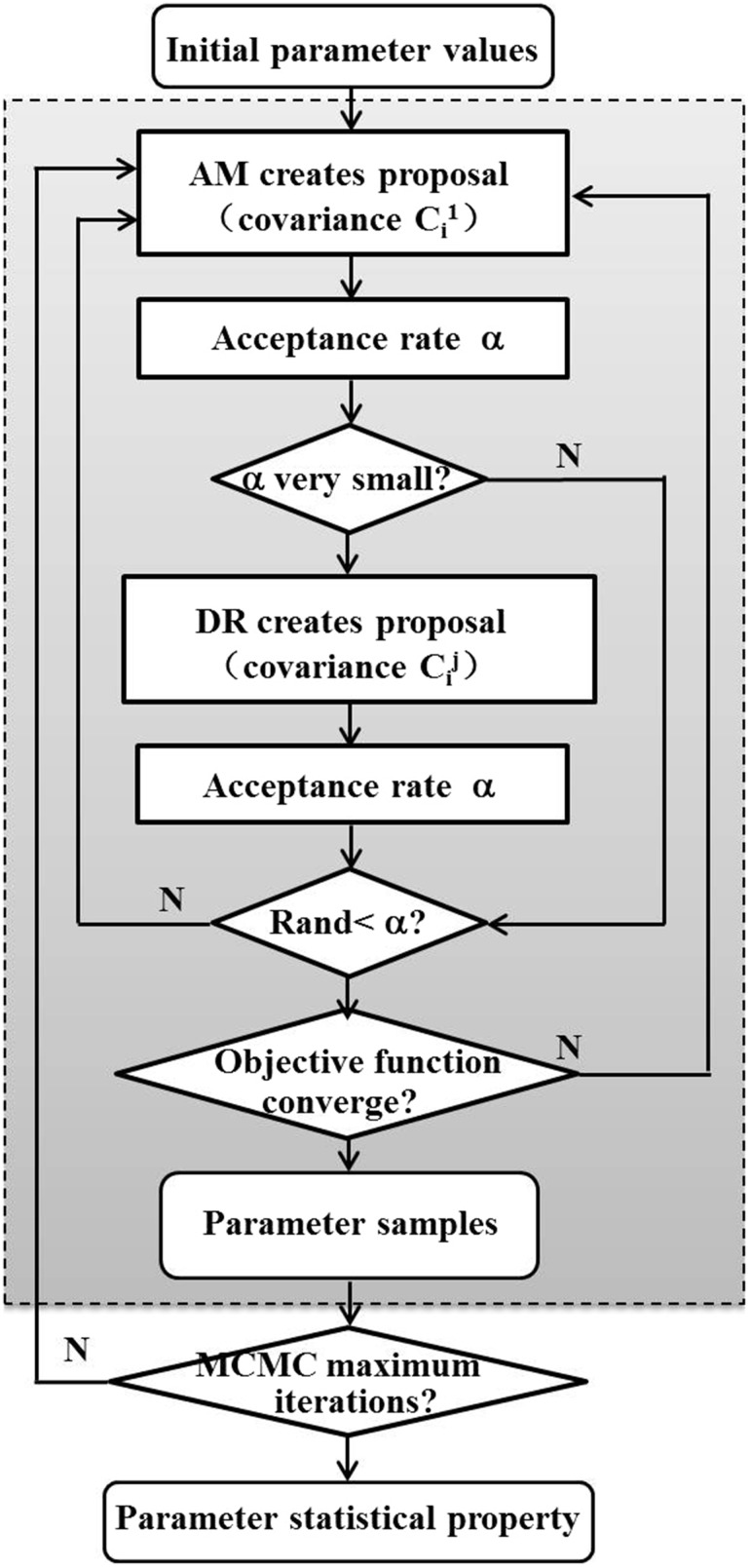
Flow chart of the improved MCMC method

In conclusion, the improved MCMC method, integrating the advantages and overcoming the disadvantages of the AM and DR algorithms, can improve the practicability significantly. The flow chart of the improved MCMC method can be seen as Fig. 1.

### Exact Zoeppritz equation

To make the method proposed in this paper suitable for reservoirs with strong-contrast interfaces and long-offset ranges and avoid calculation errors brought by the approximate Zoeppritz equation, we choose the exact Zoeppritz equation to do the inversion. The equation is as follows (Aki and Richards 1980):

5 $$R_{\text{PP}} = {{\left[ {\left( {b\frac{{\cos \theta_{1} }}{{V_{{{\text{P}}1}} }} - c\frac{{\cos \theta_{2} }}{{V_{{{\text{P}}2}} }}} \right)F - \left( {a + d\frac{{\cos \theta_{1} }}{{V_{{{\text{P}}1}} }}\frac{{\cos \varphi_{2} }}{{V_{{{\text{S}}2}} }}} \right)Hp^{2} } \right]} \mathord{\left/ {\vphantom {{\left[ {\left( {b\frac{{\cos \theta_{1} }}{{V_{{{\text{P}}1}} }} - c\frac{{\cos \theta_{2} }}{{V_{{{\text{P}}2}} }}} \right)F - \left( {a + d\frac{{\cos \theta_{1} }}{{V_{{{\text{P}}1}} }}\frac{{\cos \varphi_{2} }}{{V_{{{\text{S}}2}} }}} \right)Hp^{2} } \right]} D}} \right. \kern-0pt} D}$$

where,

6 $$\begin{aligned} & \frac{{\sin \theta_{1} }}{{V_{{{\text{P}}1}} }} = \frac{{\sin \theta_{2} }}{{V_{{{\text{P}}2}} }} = \frac{{\sin \varphi_{1} }}{{V_{{{\text{S}}1}} }} = \frac{{\sin \varphi_{2} }}{{V_{{{\text{S}}2}} }} = p, \\ & a = \rho_{2} \left( {1 - 2V_{{{\text{S}}2}}^{2} p^{2} } \right) - \rho_{1} \left( {1 - 2V_{{{\text{S}}1}}^{2} p^{2} } \right),\text{ }\quad b = \rho_{2} \left( {1 - 2V_{{{\text{S}}2}}^{2} p^{2} } \right) + 2\rho_{1} V_{{{\text{S}}1}}^{2} p^{2} , \\ & c = \rho_{1} \left( {1 - 2V_{{{\text{S}}1}}^{2} p^{2} } \right) + 2\rho_{2} V_{{{\text{S}}2}}^{2} p^{2} ,\text{ }\quad d = 2\left( {\rho_{2} V_{{{\text{S}}2}}^{2} - \rho_{1} V_{{{\text{S}}1}}^{2} } \right),\text{ } \\ & E = b\frac{{\cos \theta_{1} }}{{V_{{{\text{P}}1}} }} + c\frac{{\cos \theta_{2} }}{{V_{{{\text{P}}2}} }},\text{ }\quad F = b\frac{{\cos \varphi_{1} }}{{V_{{{\text{S}}1}} }} + c\frac{{\cos \varphi_{2} }}{{V_{{{\text{S}}2}} }}, \\ & G = a - d\frac{{\cos \theta_{1} }}{{V_{{{\text{P}}1}} }}\frac{{\cos \varphi_{2} }}{{V_{{{\text{S}}2}} }},\text{ }\quad H = a - d\frac{{\cos \theta_{2} }}{{V_{{{\text{P}}2}} }}\frac{{\cos \varphi_{1} }}{{V_{{{\text{S}}1}} }}, \\ & D = EF + GHp^{2} \\ \end{aligned}$$

$R_{\text{PP}}$ is the P-wave reflection coefficient; *θ*_1_, *θ*_2_ are the incidence or reflection angle and transmission angle of P- wave, respectively, and *φ*_1_, *φ*_2_ are the reflection angle and transmission angle of SV-wave, respectively; $V_{{{\text{P}}1}} ,V_{{{\text{P}}2}} ,V_{{{\text{S}}1}} ,V_{{{\text{S}}2}}$ are the velocities of P- and SV-waves in two layers, respectively.

### Bayesian AVO inversion based on the improved MCMC method

The inverse problem in this paper can be expressed as follows:

7 $${\mathbf{d}} = f_{{\text{Zoeppritz}}} (V_{\text{P}} ,V_{\text{S}} ,\rho ) + {\mathbf{e}}$$

where ${\mathbf{d}} = [d_{1} ,d_{2} , \ldots d_{M} ]^{T}$ represents the observed seismic data; ${\mathbf{e}}$ represents independent Gaussian distribution noise; $f_{\text{Zoeppritz}}$ represents the forward equation, which is the exact Zoeppritz equation. Based on Bayesian theory, the posterior probability density distribution of unknown parameters is given by the following formula:

8 $$p(V_{\text{P}} ,V_{\text{S}} ,\rho |{\mathbf{d}}) \propto p(V_{\text{P}} ,V_{\text{S}} ,\rho )p({\mathbf{d}}|V_{\text{P}} ,V_{\text{S}} ,\rho )$$

in which $p(V_{\text{P}} ,V_{\text{S}} ,\rho )$ indicates the prior information distribution coming from core data, logging data or other sources to ensure the inverted parameters contain low-frequency components. Based on the statistical distribution of the logging P- and S-wave velocity information and the density information, we assume that $V_{\text{P}} ,V_{\text{S}}$ and *ρ* have Gaussian distribution and are also independent of each other, with means and variances $\mu_{{V_{\text{P}} }} ,\mu_{{V_{\text{S}} }} ,\mu_{\rho }$ and $\sigma_{{V_{\text{P}} }} ,\sigma_{{V_{\text{S}} }} ,\sigma_{\rho }$, respectively. So the prior distribution function is given by:

9 $$\begin{aligned} p(V_{\text{P}} ,V_{\text{S}} ,\rho ) = p(V_{\text{P}} ) \cdot p(V_{\text{S}} ) \cdot p(\rho )\text{ } & = \frac{1}{{\left( {2\pi \sigma_{{V_{\text{P}} }}^{2} } \right)^{{\frac{N}{2}}} }}{ \exp }\left( { - \sum {\frac{{\left( {V_{\text{P}} - \mu_{{V_{\text{P}} }} } \right)^{2} }}{{2\sigma_{{V_{\text{P}} }}^{2} }}} } \right)\text{ } \\ & \quad \cdot \frac{1}{{\left( {2\pi \sigma_{{V_{\text{S}} }}^{2} } \right)^{{\frac{N}{2}}} }}{ \exp }\left( { - \sum {\frac{{\left( {V_{\text{S}} - \mu_{{V_{\text{S}} }} } \right)^{2} }}{{2\sigma_{{V_{\text{S}} }}^{2} }}} } \right)\text{ } \cdot \frac{1}{{\left( {2\pi \sigma_{\rho }^{2} } \right)^{{\frac{N}{2}}} }}{ \exp }\left( { - \sum {\frac{{\left( {\rho - \mu_{\rho } } \right)^{2} }}{{2\sigma_{\rho }^{2} }}} } \right) \\ \end{aligned}$$

where *N* is the number of the inversion parameters, and $\sigma_{{V_{\text{P}} }}^{2}$,$\sigma_{{V_{\text{S}} }}^{2}$ and *σ*_*ρ*_^2^ represent the variance of the P- and S-wave velocities and the density, respectively, and can be acquired by the statistical analysis of the logging data in practice. $p\left( {{\mathbf{d}}|V_{\text{P}} ,V_{\text{S}} ,\rho } \right)$ indicates the likelihood function combining the observed seismic data and the synthesized seismic data using the inverted parameters $V_{\text{P}} ,V_{\text{S}} ,\rho$. Assuming that ${\mathbf{e}}$ has a Gaussian distribution with zero mean and $\sigma_{\text{PP}}^{{}}$ standard deviation, the likelihood function is

10 $$p\left( {{\mathbf{d}}|V_{\text{P}} ,V_{\text{S}} ,\rho } \right) = \frac{1}{{\left( {2\pi \sigma_{\text{PP}}^{2} } \right)^{{\frac{M}{2}}} }}\exp \left( { - \sum {\frac{{\left( {{\mathbf{d}} - f_{{\text{Zoeppritz}}} \left( {V_{\text{P}} ,V_{\text{S}} ,\rho } \right)} \right)^{2} }}{{2\sigma_{\text{PP}}^{2} }}} } \right)$$

in which $\sigma_{\text{PP}}^{2}$ represents the noisy variance of the P-wave seismic data. To obtain the posterior distribution $p(V_{\text{P}} ,V_{\text{S}} ,\rho |{\mathbf{d}})$, we use the improved MCMC algorithm to generate Markov chains converging to the posterior probability density distribution $p(V_{\text{P}} ,V_{\text{S}} ,\rho |{\mathbf{d}})$.

To make the Markov chains converge to the posterior probability density distribution of the P- and S-wave velocities and the density, the stationary distribution can be described as $p(V_{\text{P}} ,V_{\text{S}} ,\rho )p({\mathbf{d}}|V_{\text{P}} ,V_{\text{S}} ,\rho )$. Let

11 $$g\left( {\mathbf{m}} \right) = - \sum {\frac{{\left( {V_{\text{P}} - \mu_{{V_{\text{P}} }} } \right)^{2} }}{{2\sigma_{{V_{\text{P}} }}^{2} }}} - \sum {\frac{{\left( {V_{\text{S}} - \mu_{{V_{\text{S}} }} } \right)^{2} }}{{2\sigma_{{V_{\text{S}} }}^{2} }}} - \sum {\frac{{\left( {\rho - \mu_{\rho } } \right)^{2} }}{{2\sigma_{\rho }^{2} }}} \text{ } - \sum {\frac{{\left( {{\mathbf{d}} - f_{\text{Zoeppritz}} \left( {V_{\text{P}} ,V_{\text{S}} ,\rho } \right)} \right)^{2} }}{{2\sigma_{\text{PP}}^{2} }}}$$

where ${\mathbf{m}}$ represents the inverted parameters $V_{\text{P}} ,V_{\text{S}} ,\rho$. Therefore, the acceptance probability can be expressed as.

12 $$\alpha ({\mathbf{m}}_{i} ,{\mathbf{m}}^{*} ) = \exp \left( {\hbox{min} \left\{ {0,g({\mathbf{m}}^{*} ) - g({\mathbf{m}}_{i} )} \right\}} \right)$$

Finally, we can create the Markov chains converging to the posterior probability density distribution of the P- and S-wave velocities and the density, and based on the statistical analysis of the Markov chains, we can obtain the inversion results of the P- and S-wave velocities and the density.

## Example

### Logging data

**Fig. 2:**
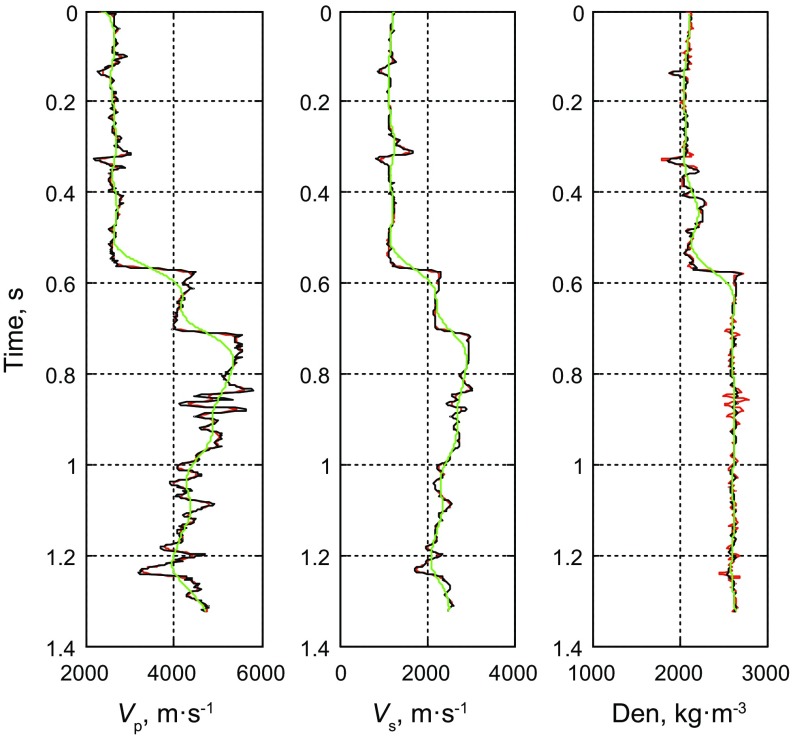
SNR = 2 results of prestack linear inversion based on the least square method using the approximate Zoeppritz equation for three parameters (*black*, *red* and *green solid lines* indicate true values, inverted results and initial model, respectively)

**Fig. 3:**
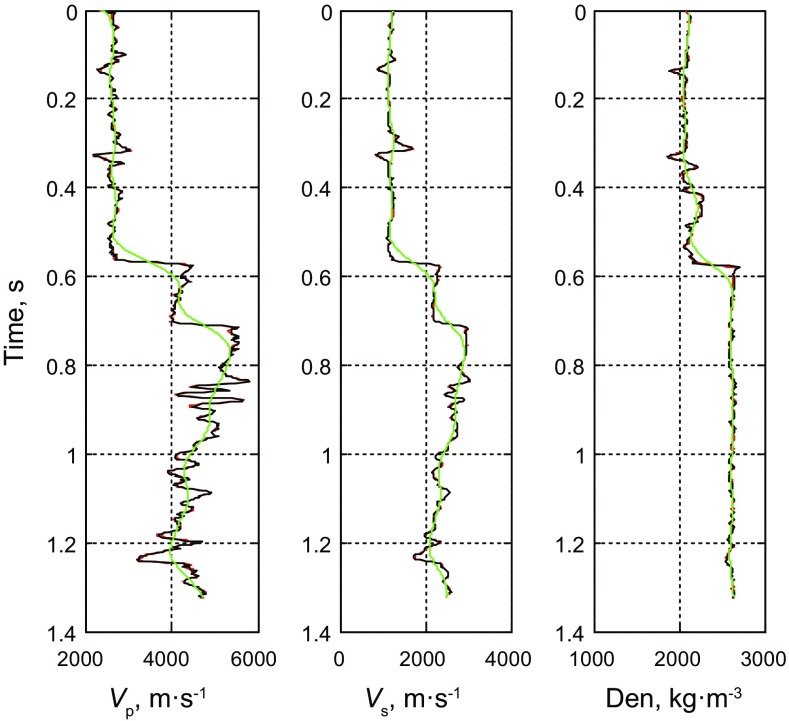
SNR = 2 results of prestack nonlinear inversion based on the improved MCMC algorithm using the exact Zoeppritz equation for three parameters (*black*, *red* and *green solid lines* indicate true values, inverted results and initial model, respectively)

We use logging data to test the feasibility of the method of prestack nonlinear inversion based on the improved MCMC algorithm using the exact Zoeppritz equation. In the forward process, we use the 35 Hz Ricker wavelet to synthetize prestack PP wave angle gathers, and add SNR = 2 (${\text{SNR}} = \frac{\sigma (d\_true)}{\sigma (d\_obs - d\_true)}$, *d*_*obs* represents the observed seismic data, and *d*_*true* represents the true synthesized data) random noise. We do the inversion using the method proposed in this paper and the method of prestack linear inversion based on the damped least square (DLS) method using the Aki and Richards (1980) approximate Zoeppritz equation to invert the P- and S-wave velocities and the density directly showed as Figs. 2 and 3, respectively. Figure 5 shows the observed seismic data for the inversion and the synthetic seismic data using the estimators.

**Fig. 4:**
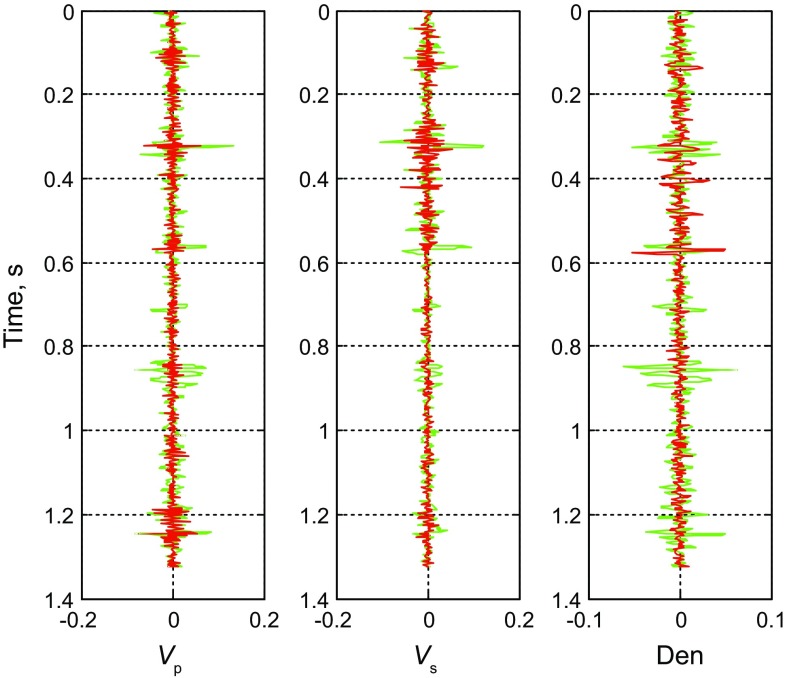
Error comparison between two methods (*green* and *red solid lines* indicate Fig. 2 results and Fig. 3 results, respectively)

**Fig. 5:**
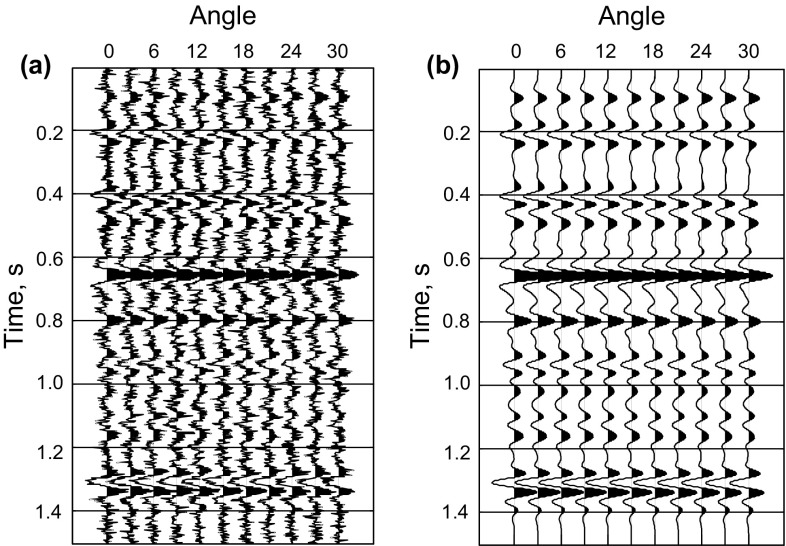
Observed seismic data and synthetic seismic data **a** the seismic data used for inversion (SNR = 2), **b** the synthetic seismic data using the estimators

From Fig. 3, we find that the inverted AVO parameters with S/N ratio of 2 are consistent with the logging data, demonstrating the effectiveness of the method. Based on the results of error comparison showed in Fig. 4, we find that both methods can obtain good inversion results, and the results of pre-stack nonlinear inversion based on the improved MCMC algorithm using the exact Zoeppritz equation have smaller errors, better stability and stronger noise immunity, so we can obtain better P- and S-wave velocities and the density, and validate the reliability and effectiveness of this method (Fig. 5).

Meanwhile, the improved MCMC inversion method can get multiple results at the same sampling point and perform probability statistics and uncertainty analysis.

**Fig. 6:**
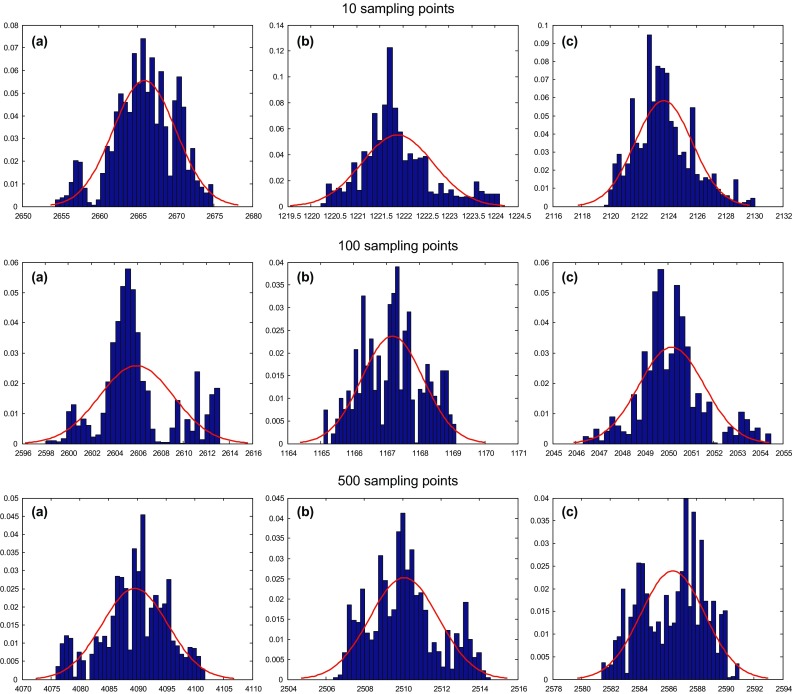
Probability statistics graphs of P- and S-wave velocities and density at 10, 100 and 500 sampling points, respectively. **a** P-wave velocity, **b** S-wave velocity, **c** density

From Fig. 6, we find that the P- and S-wave velocities and the density all show Gaussian distribution, which is consistent with the prior assumption of the three parameters, so we can use the average of the three parameters as the maximum a posteriori probability (MAP) estimation.

**Fig. 7:**
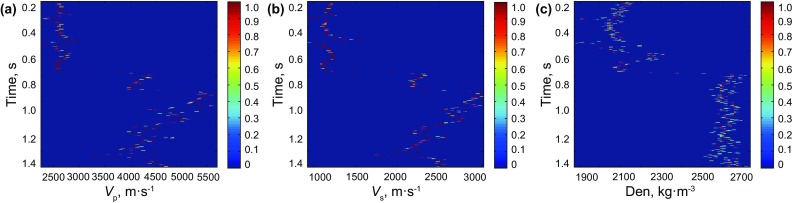
Uncertainty analysis graphs of P- and S-wave velocities and density for the logging data (*red color* indicates greater probability or smaller uncertainty). **a** P-wave velocity, **b** S-wave velocity, **c** density

The numerical magnitude in Fig. 7 represents the probability value or the uncertainty condition of the P- and S-wave velocities and the density results, and it shows that the uncertainty of the P- and S-wave velocities is smaller than that of the density, so we will further study to invert more accurate density parameters based on long-offset seismic data by using the method in this paper (Downton and Ursenbach 2006; Skopintseva et al. 2011).

### Real seismic data

**Fig. 8:**
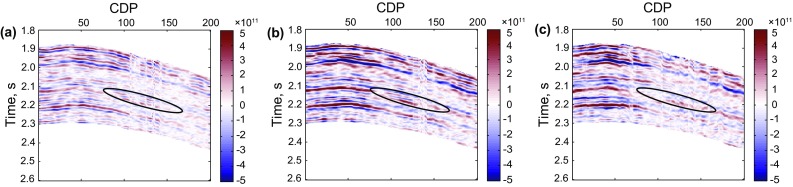
Angle-stack seismic profiles with three different angles. **a** 7° (3°–11°), **b** 15° (11°–19°), **c** 21° (19°–27°)

**Fig. 9:**
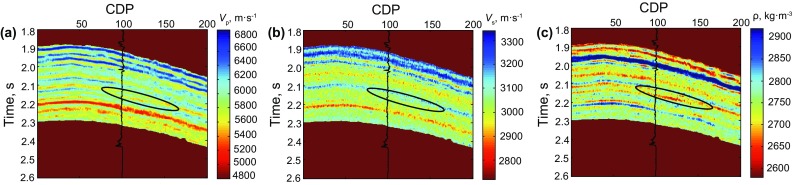
Inverted AVO parameter profiles using the improved MCMC method based on the exact Zoeppritz equation. **a** P-wave velocity profile, **b** S-wave velocity profile, **c** density profile

Real data is used to validate the application of the method of Zoeppritz-based AVO nonlinear inversion using the improved MCMC strategy. The prestack seismic data used in this paper is from an oil–gas field in the Sichuan Basin of Southwest China. Its maximum incident angle is around 27°, and the target is a carbonate gas-bearing reservoir in the Permian system. The seismic data was processed to ensure that the final prestack amplitudes should image the reflection strength of the subsurface interfaces as correctly as possible. A well located at CDP 100 (the black ellipse) shows a gas reservoir at around 2.12 s. To save the time of inversion, we stack the seismic offset gathers to three different angle-stack seismic profiles, showed as Fig. 8. The inverted results are shown as Fig. 9.

**Fig. 10:**
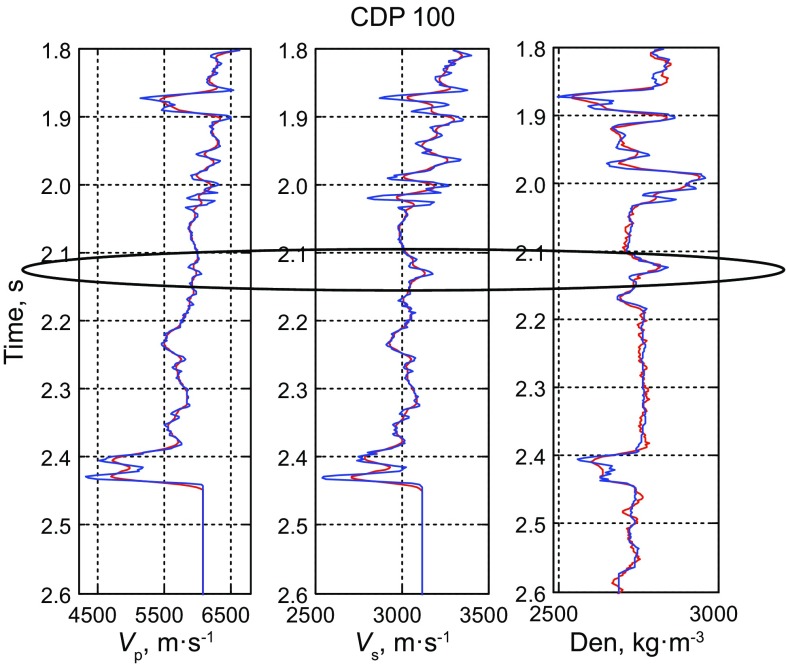
Comparison between the inversion results of near wellbore seismic trace (CDP 100) by using the improved MCMC method and the real logging data (10,000 iterations)

**Fig. 11:**
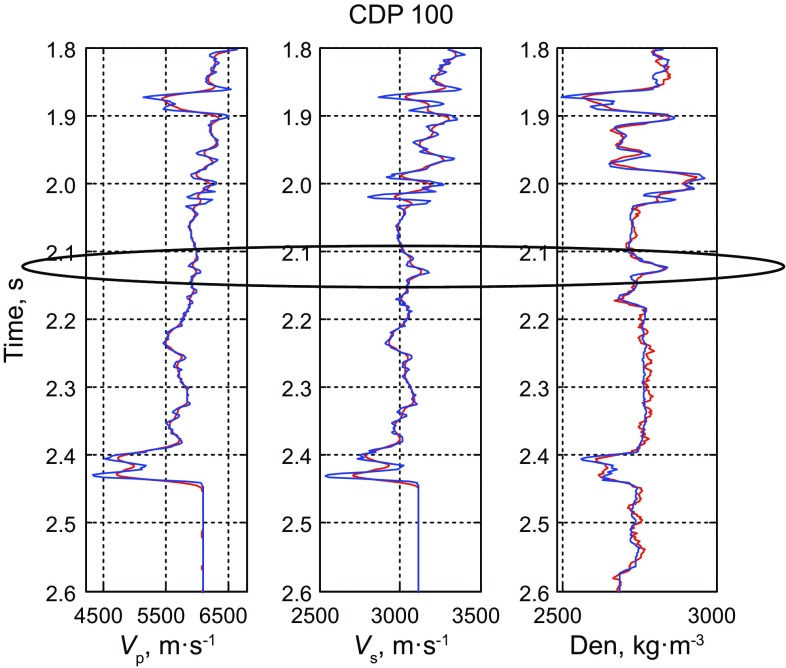
Comparison between the inversion results of near wellbore seismic trace (CDP 100) by using the conventional MCMC method and the real logging data (1,00,000 iterations)

Figure 9 shows the inverted AVO parameter profiles including P-wave velocity, S-wave velocity and density. Figure 10 shows the comparison between the inversion results and the real logging data based on the improved MCMC method, while Fig. 11 shows the comparison results based on the conventional MCMC method, where both the black ellipses in Figs. 10 and 11 indicate the location of the gas reservoir. We can see that both the inversion results of the AVO parameters fit the logging data well and they are consistent with the accuracy, but the efficiency of the improved MCMC method and the conventional MCMC method shows a great difference that the former needs 10,000 iterations while the latter needs 1,00,000 iterations to receive the results with similar accuracy. Similarly, the inversion results of P- and S-wave velocities are better than those of the density due to the limitation in the offset of the data.

## Conclusions

An improved MCMC method, combining the AM algorithm based on the global adaptive strategy and the DR algorithm based on the local adaptive strategy, has been proposed to invert the P- and S-wave velocities and the density based on the exact Zoeppritz equation. The method has the following characteristics:

1. Compared with the conventional MCMC method, the improved MCMC method, combining the AM algorithm based on the global adaptive strategy and the DR algorithm based on the local adaptive strategy, can adaptively update the proposal distribution and speed up the convergence of Markov chains;
2. The method of nonlinear inversion based on the improved MCMC algorithm using the exact Zoeppritz equation is not only suitable for reservoirs with strong-contrast interfaces and long-offset ranges but it is also more stable, accurate, and anti-noise;
3. Based on the Bayesian framework and the fusion of a priori constraint information such as logging data and seismic data, the improved MCMC method further reduces the non-uniqueness of the solutions and greatly improves the stability of the inversion solutions. Moreover, it can also estimate the uncertainty of the results to assist us in risk assessment of reservoir prediction.

Tests on logging data and seismic data demonstrate the feasibility and robustness of the method, and in order to invert more accurate density parameter, we will further study this method based on long-offset seismic data.

## References

[CR1] Aki K, Richards PG (1980). Quantitative seismology: theory and method.

[CR2] Buland A, Omre H (2003). Bayesian linearized AVO inversion. Geophysics.

[CR3] Chen TS, Wei XC. Zoeppritz-based joint AVO inversion of PP and PS waves in Ray parameter domain. In: 82th annual international meeting, SEG, Expanded Abstract. 2012. p. 1–5.

[CR4] Chen TS, Wei XC, Jia LH. Nonlinear simultaneous PP and PS AVO inversion based on Zoeppritz equations. In: Beijing 2009 international geophysical conference and exposition. 2009. p. 24–7.

[CR5] Downton JE, Ursenbach C (2006). Linearized amplitude variation with offset (AVO) inversion with supercritical angles. Geophysics.

[CR6] Du QZ, Yan HZ (2013). PP and PS joint AVO inversion and fluid prediction. J Appl Geophys.

[CR7] Fernández-Martínez JL, Fernández-Muñiz Z, Pallero JLG (2013). From Bayes to Tarantola: new insights to understand uncertainty in inverse problems. J Appl Geophys.

[CR8] Gelman A, Roberts G, Gilks W (1996). Efficient Metropolis jumping rules. Bayesian Stat.

[CR9] Green PJ, Mira A (2001). Delayed rejection in reversible jump Metropolis-Hastings. Biometrika.

[CR10] Haario H, Laine M, Mira A (2006). DRAM: efficient adaptive MCMC. Stat Comput.

[CR11] Haario H, Saksman E, Tamminen J (2001). An adaptive Metropolis algorithm. Bernoulli.

[CR12] Hong TC, Sen MK (2009). A new MCMC algorithm for seismic waveform inversion and corresponding uncertainty analysis. Geophys J Int.

[CR13] Huang HD, Wang YC, Guo F (2013). High precision pre-stack inversion algorithm based on Zoeppritz equations. OGP.

[CR14] Jilek P. Joint inversion of PP- and PS-wave reflection coefficients in anisotropic media. CWP Research Report. 2001.

[CR15] Jilek P. Joint nonlinear inversion of PP- and PS-wave reflection coefficients in anisotropic media. CWP Research Report. 2002.

[CR16] Larsen JA. AVO inversion by simultaneous P-P and P-S inversion. M.S. Dissertation, University of Calgary. 1999.

[CR17] Lehochi I, Avseth P, Hadziavidic V (2015). Probabilistic estimation of density and shear information from Zeoppritz’s equation. Lead Edge.

[CR18] Liu FP, Meng XJ, Wang YM (2010). Jacobian matrix for the inversion of P- and S-wave velocities and its accurate computation method. Sci China Earth Sci.

[CR19] Liu FP, Meng XJ, Wang YM (2012). Multi-angle inversion of formation densities based on the accurate solution of Zoeppritz’s partial derivative equations. Chin J Geophys.

[CR20] Liu FP, Meng XJ, Xiao JQ (2012). Applying accurate gradients of seismic wave reflection coefficients (SWRC) to the inversion of seismic wave velocities. Sci China Earth Sci.

[CR21] Liu HX, Li JY, Chen XH, et al. Joint AVO inversion of PP and PS reflections using the reflectivity method. In: 76th EAGE conference & exhibition. 2014.

[CR22] Lu J, Yang Z, Wang Y (2015). Joint PP and PS AVA seismic inversion using exact Zoeppritz equations. Geophysics.

[CR23] Rabben TE, Tjelmeland H, Ursin B (2008). Non-linear Bayesian joint inversion of seismic reflection coefficients. Geophys J Int.

[CR24] Russell BH, Gray D, Hampson DP (2011). Linearized AVO and poroelasticity. Geophysics.

[CR25] Skopintseva L, Ayzenberg M, Landrø M (2011). Long-offset AVO inversion of PP reflections from plane interfaces using effective reflection coefficients. Geophysics.

[CR26] Tian YK, Zhou H, Yuan SY (2013). Lithologic discrimination method based on Markov random-field. Chin J Geophys.

[CR27] Wang DY, Zhang GZ. AVO simultaneous inversion using Markov Chain Monte Carlo. In: 80th annual international meeting, SEG, Expanded Abstract. 2010. p. 444–48.

[CR28] Wang FF, Li JY, Chen XH (2014). Bayesian facies identification based on Markov-chain prior model. OGP.

[CR29] Wang YM, Wang XP, Meng XJ, et al. Pre-stack inversion of wide incident angle seismic data. In: 81th annual international meeting, SEG, Expanded Abstract. 2011. p. 2507–11.

[CR30] Yang PJ, Wang CJ, Bi JF (2015). Direct extraction of the fluid factor based on variable point-constraint. Chin J Geophys.

[CR31] Yin XY, Zhang SX, Zhang F (2013). Two-term elastic impedance inversion and Russell fluid factor direct estimation method for deep reservoir fluid identification. Chin J Geophys.

[CR32] Yin XY, Zong ZY, Wu GC (2015). Research on seismic fluid identification driven by rock physics. Sci China Earth Sci.

[CR33] Zhang FQ, Wei FJ, Wang YC (2013). Generalized linear AVO inversion with the priori constraint of trivariate Cauchy distribution based on Zoeppritz equation. Chin J Geophys.

[CR34] Zhang GZ, Du BY, Li HS (2014). The method of joint pre-stack inversion of PP and P-SV waves in shale gas reservoirs. Chin J Geophys.

[CR35] Zhang GZ, Wang DY, Yin XY (2011). Study on prestack seismic inversion using Markov Chain Monte Carlo. Chin J Geophys.

[CR36] Zhang Z, Yin XY, Hao QY (2014). Frequency-dependent fluid identification method based on AVO inversion. Chin J Geophys.

[CR37] Zhi LX, Chen SQ, Li XY. Joint AVO inversion of PP and PS waves using exact Zoeppritz equation. In: 83th annual international meeting, SEG, Expanded Abstract. 2013. p. 457–61.

[CR38] Zhi LX, Chen SQ, Li XY, et al. An improved strategy for exact Zoeppritz equations AVA inversion. In: 85th annual international meeting, SEG, Expanded Abstract. 2015. p. 654–58.

[CR39] Zhu XF, McMechan G. AVO inversion using the Zoeppritz equation for PP reflections. In: 82th annual international meeting, SEG, Expanded Abstract. 2012. p. 1–5.

[CR40] Zong ZY, Yin XY, Wu GC (2012). AVO inversion and poroelasticity with P- and S-wave moduli. Geophysics.

[CR41] Zong ZY, Yin XY, Wu GC (2013). Elastic impedance parameterization and inversion with Young’s modulus and Poisson’s ratio. Geophysics.

[CR42] Zong ZY, Yin XY, Wu GC (2013). Multi-parameter nonlinear inversion with exact reflection coefficient equation. J Appl Geophys.

[CR43] Zunino A, Mosegaard K, Lange K (2015). Monte Carlo reservoir analysis combining seismic reflection data and informed priors. Geophysics.

